# Co-activation of PIK3CA and Yap promotes development of hepatocellular and cholangiocellular tumors in mouse and human liver

**DOI:** 10.18632/oncotarget.3546

**Published:** 2015-03-12

**Authors:** Xiaolei Li, Junyan Tao, Antonio Cigliano, Marcella Sini, Julien Calderaro, Daniel Azoulay, Chunmei Wang, Yan Liu, Lijie Jiang, Katja Evert, Maria I. Demartis, Silvia Ribback, Kirsten Utpatel, Frank Dombrowski, Matthias Evert, Diego F. Calvisi, Xin Chen

**Affiliations:** ^1^ Department of Hepatobiliary Surgery, Xijing Hospital, The Fourth Military Medical University, Xi'an, Shaanxi, P.R. China; ^2^ Department of Bioengineering and Therapeutic Sciences and Liver Center, University of California, San Francisco, CA, U.S.A; ^3^ School of Pharmacy, Hubei University of Chinese Medicine, Wuhan, Hubei, P.R. China; ^4^ Institute of Pathology, University Medicine of Greifswald, Greifswald, Germany; ^5^ Department of Pathology, Assistance Publique-Hôpitaux de Paris, Centre Hospitalier Universitaire Henri Mondor, Créteil, France; ^6^ Inserm, U1162, Génomique Fonctionnelle des Tumeurs Solides, Institut Universitaire d'Hematologie, Paris, France; ^7^ Department of Digestive and Hepatobiliairy Surgery, Assistance Publique-Hôpitaux de Paris, Centre Hospitalier Universitaire Henri Mondor, Créteil, France; ^8^ Department of Clinical and Experimental Medicine, University of Sassari, Sassari, Italy

**Keywords:** HCC, cholangiocarcinoma, liver tumor, PI3K, hippo

## Abstract

Activation of the PI3K and Yes-associated protein (Yap) signaling pathways has been independently reported in human hepatocellular carcinoma (HCC). However, the oncogenic interactions between these two cascades in hepatocarcinogenesis remain undetermined. To assess the consequences of the crosstalk between the PI3K and Yap pathways along liver carcinogenesis, we generated a mouse model characterized by combined overexpression of activated mutant forms of PIK3CA (PIK3CAH1047R) and Yap (YapS127A) in the mouse liver using hydrodynamic transfection (PIK3CA/Yap). In addition, suppression of PI3K and Yap pathways was conducted in human HCC and cholangiocarcinoma (CCA) cell lines. We found that concomitant activation of PI3K and Yap pathways triggered rapid liver tumor development in mice. Histologically, tumors were pure HCC, CCA, or mixed HCC/CCA. At the molecular level, PIK3CA/Yap tumors were characterized by activation of the mTORC1/2, ERK/MAPK, and Notch pathways. Simultaneous activation of PI3K and Yap pathways frequently occurred in human liver tumor specimens and their combined suppression was highly detrimental for the growth of HCC and CCA cell lines. In conclusion, our study demonstrates the oncogenic cooperation between PI3K and Yap pathways along liver carcinogenesis. The PIK3CA/Yap mouse represents an important preclinical liver tumor model for the development of novel therapeutics against this malignancy.

## INTRODUCTION

Primary liver cancer is one of the most common malignancies in adults and a leading cause of cancer related deaths worldwide [1]. Hepatocellular carcinoma (HCC) and cholangiocarcinoma (CCA) are the major types of primary liver cancer, accounting for almost 90% and ~10% of all liver tumors, respectively [2]. Another liver tumor entity, known as mixed HCC/CCA, can also occur, although at significantly lower frequency [3]. Due to its increasing incidence and related poor survival [1], innovative therapeutic options for liver cancer patients are necessary. For this purpose, a deeper knowledge of the molecular mechanisms underlying liver cancer development is highly required.

Yap is a major downstream effector of the Hippo pathway, an evolutionally well-conserved potent regulator of organ size, tissue regeneration, stem cell self-renewal, and tumor development [4, 5]. Inhibition of the Hippo tumor suppressor cascade promotes dephosphorylation of Yap, leading to its nuclear localization. Once in the nucleus, Yap interacts with a number of transcription factors to induce the expression of target genes, such as CTGF, Cyr61, and Survivin, whose upregulation promotes cellular proliferation and survival [4, 5]. The Yap serine 127 to alanine (S127A) mutant is a constitutively active form that remains in the nucleus and is transcriptionally active [6]. Recently, numerous studies have detected overexpression of Yap in a variety of human tumor types, including colorectal, ovarian, and lung cancer [7]. In HCC, Yap has been identified as a driver oncogene [8] and an independent factor in predicting poor disease-free and overall survival [9].

The phosphatidylinositol 3-kinase/mammalian target of rapamycin (PI3K/mTOR) pathway is aberrantly activated in variable types of cancers [10, 11], including in 30-50% of HCC cases [12, 13]. This signaling cascade plays a pivotal role in many cellular processes, including growth, proliferation, survival, autophagy, metabolism, and cytoskeletal organization [14, 15]. PI3Ks are heterodimeric lipid kinases composed of p110 catalytic subunits and p85 regulatory subunits, interacting with phosphatidylinositol-3-phosphate at the membrane and catalyzing the phosphorylation of AKT [16]. Mutant forms of PIK3CA, which encodes the p110a catalytic subunit, have been found in various human cancers, including colon, breast, lung cancer, and HCC, leading to increased lipid kinase activity and oncogenesis [17-20].

Both Yap and PI3K/mTOR signaling pathways are potent inducers of hepatocarcinogenesis, and a previous study showed that activation of Yap and PI3K/AKT/mTOR signaling correlated positively in HCC [21]. However, whether the two pathways functionally interact along hepatocarcinogenesis has not been investigated to date, especially *in vivo*. Here, we show that concomitant activation of PI3K and Yap cascades in mice promotes rapid development of liver tumors characterized by hepatocellular and/or cholangiocellular features. Furthermore, we found that the two pathways are frequently activated in human HCC and cholangiocarcinoma (CCA) as well as in mixed HCC/CCA samples. Thus, therapeutic approaches aimed at suppressing the PI3K and Yap pathways might be highly beneficial for the treatment of liver tumors with both hepatocellular and cholangiocellular features.

## RESULTS

### Concomitant activation of PI3K and Yap1 led to rapid liver tumor development in mice

**Figure 1:**
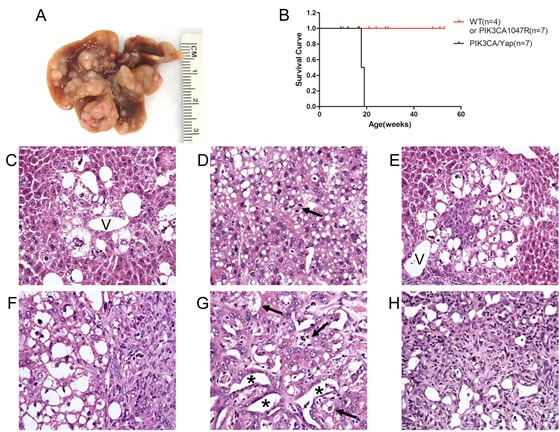
Histologic features of liver tumors developed in PIK3CA/Yap mice as assessed by H&E staining (A) Macroscopic appearance of livers from mice injected wih PIK3CAH1047R and YapS127A (PIK3CA/Yap) mice. (B) Kaplan Meier survival curve of wild-type (WT), PIK3CA1047R and PIK3CA/Yap mouse cohort. (C) Preneoplastic lesion consisting of lipid-rich ballooned hepatocytes located around a hepatic vein (V). (D) Pure hepatocellular carcinoma (HCC) characterized by solid and trabecular growth of mildly atypical lipid-rich neoplastic hepatocytes. The arrow indicates a mitosis. (E) Small mixed tumor consisting of both hepatocellular and cholangiocellular components. The hepatocellular part of the tumor consists of large, lipid-rich cells, mainly situated in the outer part of the tumor. Smaller cells with a high nuclear:cytoplasmic ratio, located in the core of the lesion, constitute the cholangiocellular part of the tumor. (F) Mixed HCC/cholangiocarcinoma (CCA) tumor displaying the presence of the hepatocellular component (left part of the picture) that is adjacent to the cholangiocellular component (right part of the picture). (G) Mixed HCC/CCA tumor with hepatocellular and cholangiocellular constituents intermingled with each other, showing moderate cellular atypia and a limited stroma component. The cholangiocellular component forms duct-like structures (asterisks), whereas the hepatocellular component consists of altered, lipid-rich hepatocytes (indicated by arrows). (H) Pure CCA showing significant cellular atypia. Original magnification: 400X.

To investigate the genetic crosstalk between the PI3K and Yap signaling pathways *in vivo*, we overexpressed commonly activated mutant forms of PIK3CA (PIK3CAH1047R, with a HA tag) and Yap1 (YapS127A, with a Flag tag) in the mouse liver using hydrodynamic transfection. As we reported previously [22], overexpression of YapS127A alone did not result in any liver anomaly even after 22.5 weeks post injection (data not shown), whereas overexpression of PIK3CAH1047R (which will be referred to as PIK3CA mouse) resulted in the occurrence of lipid-rich hepatocytes forming clusters throughout the liver parenchyma. Morphologically, these hepatocytes resembled the altered hepatocytes occurring in the livers of mice injected with the myristylated/activated form of AKT1 [23]. However, no tumors developed up to 40 weeks post injection in PIK3CA mice (Supplementary Figure 1A-C). A more detailed description of PIK3CAH1047R mice will be presented elsewhere. In striking contrast, co-expression of PIK3CAH1047R and YapS127A (which will be referred to as PIK3CA/Yap mouse) led to rapid liver tumor development within 12-13 weeks post injection (Figure 1A and B). At the histological level, ~80% of the liver parenchyma from PIK3CA/Yap mice was occupied by tumor lesions, with the remaining liver tissue consisting of lipid-rich hepatocytes (morphologically identical to those detected in PIK3CA mice) and normal liver tissue (Figure 1C-H). Of note, three distinct tumor types were detected in PIK3CA/Yap mice: (i) pure hepatocellular (~40% of all detected tumors), characterized by a solid or macrotrabecular growth pattern, often accompanied by cytoplasmic lipid accumulation and mild cytological atypia; (ii) pure cholangiocellular (~10%), forming primitive ducts and exhibiting stromal reaction; (iii) mixed HCC/CCC (~50% of the tumor lesions), with a hepatocellular component and the other component consisting of spindle-like or oval-cell-like small basophilic cells that resembled CCA-cells (although only rarely forming ductular structures and often lacking desmoplastic stroma) (Figure 1C-H). While pure hepatocellular tumors were generally well-differentiated and rarely showed areas of moderate cellular atypia or necrosis, mixed HCC/CCA and pure cholangiocellular tumors displayed diffuse and moderate to severe atypia. As multiple tumors developed in each PIK3CA/Yap mouse, the three distinct tumor types were detected in each animal investigated.

**Figure 2:**
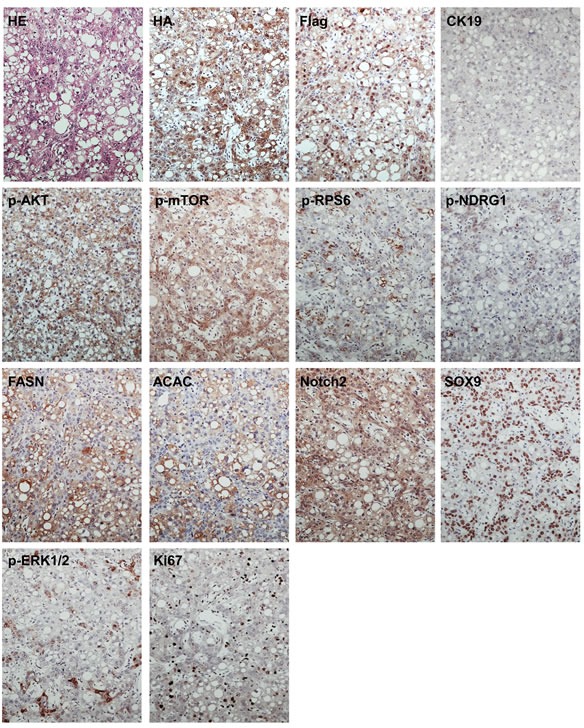
Molecular characterization of hepatocellular tumors developed in PIK3CA/Yap mice These tumors are homogeneously immunoreactive for HA-tagged PIK3CAH1047R (HA) and Flag-tagged Yap (Flag), implying their origin from doubly-transfected cells. The low/absent immunoreactivity for CK19 confirms their hepatocellular nature. These tumors exhibit strong activation of PI3K/AKT/mTOR pathway, as indicated by levels of phosphorylated/activated (p-AKT) and its downstream effectors, including phosphorylated/activated mTOR (p-mTOR), fatty acid synthase (FASN), acetyl-CoA carboxylase (ACAC), and phosphorylated/activated ribosomal protein S6 (p-RPS6), whereas immunolabeling for phosphorylated N-Myc downregulated gene 1 (p-NDGR1), a surrogate marker of mTORC2 activation, is weak. Hepatocellular tumors also displayed a remarkable, homogeneous activation of the Notch cascade, as indicated by the strong immunoreactivity for Notch2 and its downstream effector, SOX9. In addition, these tumors showed activation of Ras/MAPK pathway, as underscored by spotty immunolabeling for phosphorylated/activated ERK1/2 (p-ERK1/2) proteins. The moderate proliferative activity of these lesions is indicated by positive immunolabeling for Ki67. Serial sections of a hepatocellular tumor are shown as an example in the present figure. Original magnification: 100X Abbreviation: HE, hematoxylin and eosin staining.

**Figure 3:**
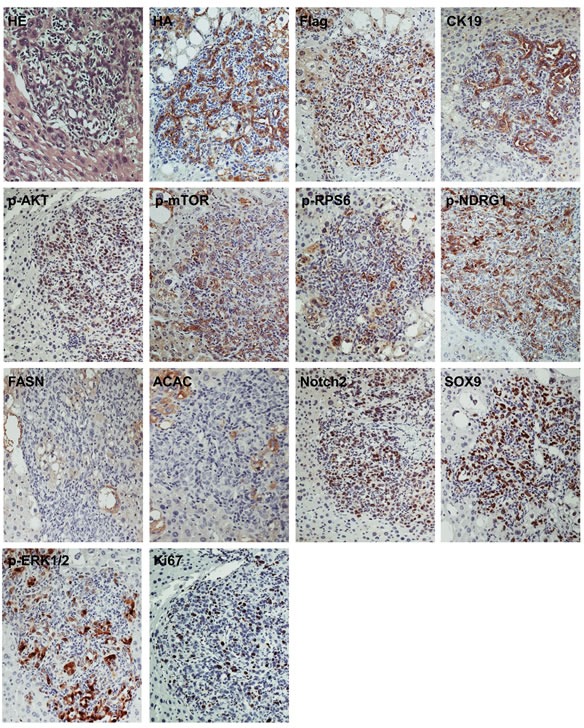
Molecular characterization of cholangiocellular tumors developed in PIK3CA/Yap mice Similar to pure hepatocellular carcinomas (HCC) developed in these mice, cholangiocellular tumors are homogeneously immunoreactive for HA-tagged PIK3CAH1047R (HA) and Flag-tagged Yap (Flag), implying their origin from doubly-transfected cells. The strong immunoreactivity for CK19 confirms their cholangiocellular differentiation. These tumors exhibit strong activation of PI3K/AKT/mTOR pathway, as indicated by elevated levels of phosphorylated/activated (p-AKT) and its downstream effectors, including phosphorylated/activated mTOR (p-mTOR) and phosphorylated N-Myc downregulated gene 1 (p-NDGR1), a surrogate marker of mTORC2 activation, whereas immunoreactivity for fatty acid synthase (FASN), acetyl-CoA carboxylase (ACAC), and phosphorylated/activated ribosomal protein S6 (p-RPS6) is limited. These tumors also displayed a marked activation of the Notch cascade, as indicated by the strong immunolabeling for Notch2 and its downstream effector, SOX9. The activation of the Ras/MAPK pathway is underscored by the immunoreactivity for phosphorylated/activated ERK1/2 (p-ERK1/2) proteins, while the proliferative activity of these lesions is indicated by the positive immunolabeling for Ki67. Serial sections of a cholangiocellular tumor are shown as an example in the present figure. Original magnification: 200X. Abbreviation: HE, hematoxylin and eosin staining.

**Figure 4:**
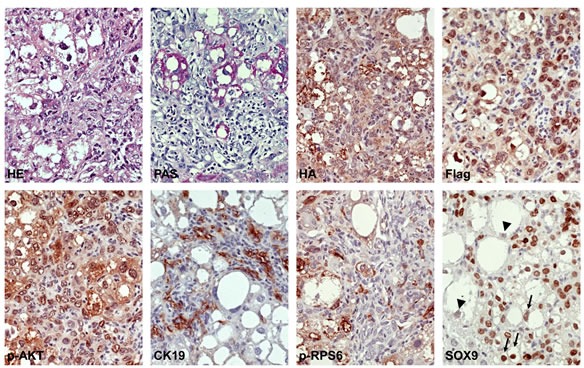
Molecular features of mixed hepatocellular/cholangiocellular tumors developed in PIK3CA/Yap mice The hepatocellular and cholangiocellular components are depicted in the left and right part of the pictures, respectively. Note that the hepatocellular component is rich in glycogen, as indicated by the PAS reaction, whereas the cholangiocellular counterpart is depleted of glycogen. These tumors are homogeneously immunoreactive for HA-tagged PIK3CA1047R (HA) and Flag-tagged Yap (Flag), indicating their origin from doubly-transfected cells. As expected, CK19 immunolabeling was detected only in the cholangiocellular fraction of the mixed tumor. Of note, levels of phosphorylated/activated ribosomal protein S6 (p-RPS6) were elevated only in the hepatocellular component, whereas immunoreactivity for SOX9 (a marker of Notch cascade activation) was evident both in the hepatocellular and cholangiocellular component. While the malignant cells with cholangiocellular features were homogeneously positive for SOX9, the hepatocellular neoplastic component consisted of cells with moderate to strong immunoreactivity (arrows) and others with weak immunolabeling (arrowheads) for this protein. Original magnification: 400X Abbreviation: HE, hematoxylin and eosin staining; PAS, periodic acid-Schiff reaction.

To confirm that the observed tumors were indeed induced by the ectopically injected oncogenes, immunohistochemistry (IHC) was performed in preneoplastic and neoplastic lesions from PIK3CA/Yap mice using an anti-HA and anti-Flag-tag antibody, which indicate the expression of PIK3CACH1047R and activated Yap, respectively. As expected, strong expression of HA-tag and Flag-tag was detected in preneoplastic (not shown) and neoplastic lesions from PIK3CA/Yap mice 12 weeks post injection (Figures 2, 3, 4; Supplementary Figure 2).

### PIK3CA/Yap co-expression promotes activation of the AKT/mTOR, ERK/MAPK, and Notch pathways in the mouse liver

To elucidate the molecular mechanisms mediating tumor development in PIK3CA/Yap injected mice, we analyzed the key downstream signaling pathways of PI3K and Yap cascades in preneoplastic and neoplastic liver lesions from PIK3CA/Yap mice by immunohistochemistry (Figures 2, 3, 4; Supplementary Figure 2; Supplementary Table 2). In hepatocellular lesions (Figure 2; Supplementary Figure 2), a strong activation of the PI3K/AKT/mTOR cascade was underscored by elevated levels of phosphorylated/activated (p)-AKT and mTOR as well as by increased levels of mTORC1 targets, namely phosphorylated/activated (p)-ribosomal protein S6 (p-RPS6), fatty acid synthase (FASN) and acetyl-CoA carboxylase (ACAC), whereas low immunoreactivity was detected for the surrogate marker of mTORC2 activity, phosphorylated N-Myc donwregulated gene 1 (NDRG1). Next, since in a previous study we showed that YAP induces the Notch pathway in HCC cells and mouse hepatocytes [24], we evaluated the activation of the Notch cascade. Importantly, we found high levels of Notch2 and its target, SOX9, in PIK3CA/Yap preneoplastic and neoplastic lesions, indicating the activation of Notch pathway along hepatocarcinogenesis in these mice. In addition, spotty immunolabeling for phosphorylated/activated (p-) ERK1/2 proteins was detected in hepatocellular lesions from PIK3CA/Yap mice. In cholangiocellular lesions, the same immunohistochemical pattern was detected for most of the proteins tested, with few exceptions. Indeed, immunoreactivity for p-RPS6 and lipogenic proteins (FASN, ACAC) was lower than in hepatocellular lesions, whereas cholangiocellular lesions exhibited a more pronounced immunolabeling for p-NDRG1 and p-ERK1/2 (Figure 3). The same immunohistochemical differences were maintained in mixed hepatocellular/cholangiocellular lesions (Figure 4; Supplementary Figures 2 and 3). Very faint or absent staining for the same proteins was detected in livers from mice injected with the empty vector or wild-type livers (Supplementary Figure 4). A summary of the immunostaining patterns of the various proteins tested is depicted in Supplementary Figure 2 and Supplementary Table 2.

### Transcriptionally active Yap is required for hepatocarcinogenesis in association with PIK3CA

**Figure 5:**
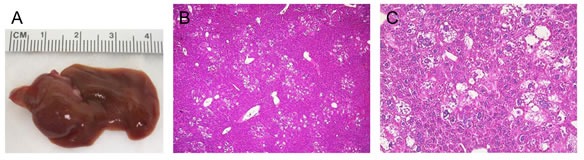
Combined injection of PIK3CAH1047R and a mutant form of Yap that does not bind TEAD transcription factors (YapS127AS94A) abolishes tumor development in mice (A) Macroscopic appearance of livers from PIK3CA/YapS127AS94A mice 13 weeks post hydrodynamic injection. (B and C) Lower (B) and higher (C) magnification of PIK3CAH1047R/YapS127AS94A mouse liver showing the absence of tumors, as assessed by Hematoxylin and eosin staining. At higher magnification, the presence of lipid-rich hepatocytes resembling those occurring in mice injected only with PIK3CAH1047R is appreciable. Original magnification: 40X in B; 200X in C.

As a transcriptional co-activator, YAP functions via interacting with a panel of transcription factors, such as TEAD family members [25] and SMADs to promote gene expression [26]. To determine whether TEADs are the transcription factors required for PIK3CA/Yap induced tumors, the binding site for TEAD genes was mutated in the constitutively active Yap gene (YapS127AS94A) [25]. Of note, combined injection of PIK3CAH1047R and YapS127AS94A resulted in the complete abrogation of tumor development in mice 13 weeks post injection (Figure 5A-C). Indeed, at this time point, mice injected with PIK3CAH1047R and YapS127AS94A were characterized by the presence of lipid-rich hepatocytes that were identical to those detected in PIK3CA mice. This finding indicates that the interaction of Yap with TEAD transcription factors is critical to cooperate with PI3K to induce liver tumors in mice.

### Levels of PIK3CA and Yap are often concomitantly elevated in human HCC, CCA, and mixed HCC/CCA specimens

**Figure 6:**
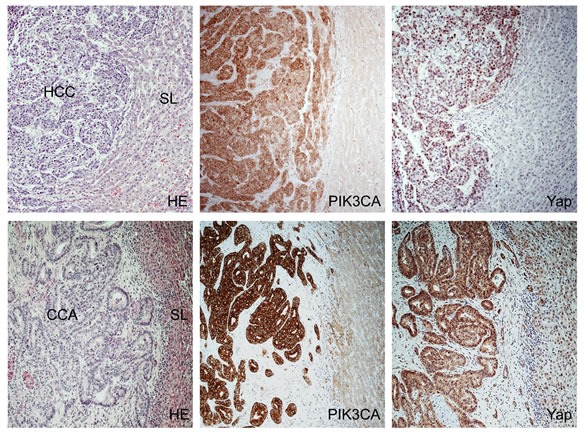
Immunohistochemical patterns of PIK3CA and Yap in human hepatocellular carcinoma (HCC) and cholangiocarcinoma (CCA) Upper panel: stronger immunoreactivity for PIK3CA as well as upregulation of Yap and its increased nuclear accumulation in a HCC (left part of the picture) with a pseudoglandular phenotype when compared to the non-neoplastic surrounding liver (SL; right part of the picture). Lower panel, upregulation of PIK3CA and total and nuclear levels of Yap in a CCA (left part of the figure) when compared with the non-tumorous counterpart (right part of the picture). Abbreviations: CCA, cholangiocarcinoma; HCC, hepatocellular carcinoma; HE, hematoxylin and eosin staining; SL, surrounding liver. Original magnification: 100X.

**Figure 7:**
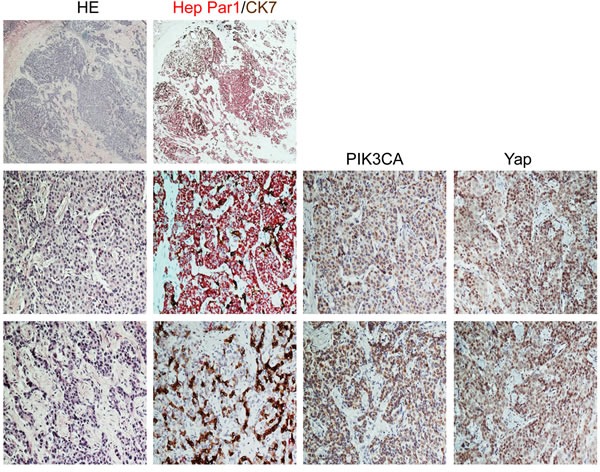
Immunohistochemical patterns of PIK3CA and Yap in a mixed human hepatocellular carcinoma (HCC)/cholangiocarcinoma (CCA) Upper panel: the tumor exhibits areas with strong immunoreactivity for Hep Par1 (a hepatocellular marker, stained in red) intermingled with other areas positive for CK7 (a cholangiocellular marker, stained in brown) immunohistochemistry. Middle panel: Area of the mixed human HCC/CCA with mainly hepatocellular differentiation (as indicated by the large prevalence of Hep Par1 positive cells over those displaying CK7 immunoreactivity) shows strong immunolabeling for both PIK3CA and Yap proteins, with the latter mainly localized in the nucleus of malignant cells. Lower panel: Area of the same tumor with predominant cholangiocellular featues (as demonstrated by the strong CK7 staining) exhibiting a homogeneous and pronounced immunoreactivity for PIK3CA and Yap proteins. Abbreviations: CCA, cholangiocarcinoma; HCC, hepatocellular carcinoma; HE, hematoxylin and eosin staining. Original magnification: 20X in upper panel; 200X in middle and lower panel.

Next, we evaluated the possible relationship between PI3K and Yap pathways in human liver cancer. For this purpose, we analyzed a collection of human HCC (n=54), CCA (n=42), and mixed HCC/CCA (n=16) by immunohistochemistry for PIK3CA and Yap staining (Figures 6 and 7). In HCC, upregulation of PIK3CA and nuclear accumulation of Yap were detected in 15 (27.8%) and 38 (70.4%) specimens, respectively. Importantly 9 of 15 (60%) HCC specimens showing upregulation of PIK3CA concomitantly exhibited nuclear localization of the Yap protein (Figure 6). In CCA, PIK3CA levels were induced in 24 of 42 (57.1%) samples, whereas all specimens displayed immunoreactivity for Yap (Figure 6), in accordance with previous reports [22]. Finally, PIK3CA levels were elevated in 11 of 16 (68.8%) mixed HCC/CCA, while Yap nuclear translocation was detected in 14 of the latter specimens (87.8%). Simultaneous upregulation of PIK3CA and nuclear localization of Yap occurred in 10 of 16 (62.5%) mixed HCC/CCA (Figure 7). No association between the staining patterns of PIK3CA and Yap and clinicopathological features of the HCC, CCA, and mixed HCC/CCA patients, including etiology, presence of cirrhosis, α-fetoprotein levels, tumor size, and tumor grading was found (data not shown). Altogether, the present data indicate that induction of PIK3CA and activation of Yap often co-exist in human liver tumors with hepatocellular and cholangiocellular features.

### Combined suppression of PIK3CA and Yap pathways is highly detrimental for the growth of human HCC and CCA cell lines

**Figure 8:**
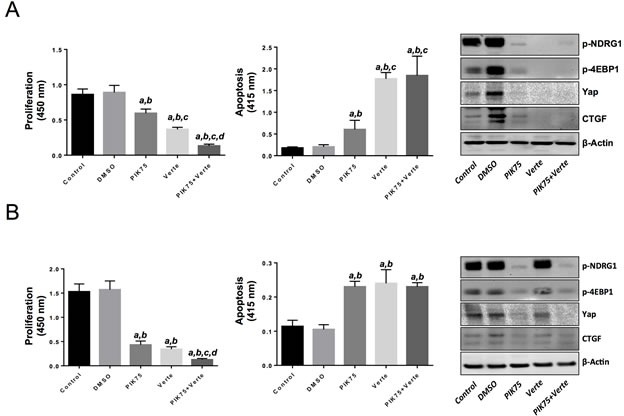
Suppression of PIK3CA and Yap activity via specific inhibitors is highly detrimental for the growth of human HLF hepatocellular carcinoma (HCC) cell line and the human EGI1 cholangiocarcinoma (CCA) cell line (A) Treatment with the PIK3CA inhibitor, PIK75 (1 μM), or the Yap/TEAD disruptor, Verteporfin (Verte; 2 μM) decreased proliferation (left panel) and induced apoptosis (center panel) in the HLF HCC cell line when compared with control (untreated) and DMSO (solvent) treated cells. Of note, combined administration of PIK75 and Verteporfin further decreased the proliferation rate of HLF cells without further augmenting apoptosis. The effects of PIK75 and Verteporfin treatment on PIK3CA targets (phosphorylated-NDRG1 and phosphorylated/inactivated 4EBP1) as wells as on Yap and its effector, CTGF, in HLF cells were assessed by Western blot analysis (right panel). (B) A similar growth restraint patterns as those described in (A) was also detected when the EGI1 CCA cell line was subjected to the administration of the two inhibitors, either alone or in combination. Once again, the additive effects of the two drugs affected only the proliferation rate but not the apoptosis activity in EGI1 cells. The effects of PIK75 and Verteporfin treatment on PIK3CA targets (phosphorylated-NDRG1 and phosphorylated/inactivated 4EBP1) as wells as on Yap and its effector, CTGF, in EGI1 cells were assessed by Western blot analysis (right panel). Each bar represent mean ± SD of three independent experiments conducted in triplicate. Tukey-Kramer's test: P at least < 0.001 *a*, versus control (untreated cells); *b*, versus DMSO (solvent); *c,* versus PIK75; *d*, versus Verteporfin. Abbreviation: Verte, Verteporfin.

Finally, we assessed the importance of the PI3K and Yap on the *in vitro* growth of human HCC and CCA cell lines. For this purpose, the PIK3CA specific inhibitor, PIK75 [27], and the disruptor of Yap-TEAD interaction, Verteporfin [28], were applied either alone or in combination in HLF and SK/Hep1 HCC cell lines and the EGI1 CCA cell line (Figure 8A and B, Supplementary Figure 5). Treatment with the two inhibitors alone resulted in a strong decrease of proliferation and induction of apoptosis in the three cell lines. A further reduction of proliferation was detected in the three cell lines when the two drugs were administered combinatorially, whereas no additive effects on apoptosis were observed (Figure 8A and B, Supplementary Figure 5). At the molecular level, inhibition of PIK3CA activity by PIK75 resulted, as expected, in the downregulation of PIK3CA canonical targets, such as phosphorylated NDRG1 and phosphorylated/inactivated 4EBP1 in HLF and EGI1 cell lines (Figure 8A and B). Of note, PIK75 administration was followed by decreased levels of Yap and connective tissue growth factor (CTGF), a Yap target, in both HLF and EGI1 cells (Figure 8A and 8B). Treatment with Yap/TEAD disruptor, Verteporfin, led instead to the reduction of Yap and CTGF levels in both HLF and EGI1 cell lines, whereas Verteporfin administration triggered downregulation of the PIK3CA targets, namely phosphorylated NDRG1 and phosphorylated/inactivated 4EBP1, only in HLF cells (Figure 8A and B). Altogether, the present data indicate that simultaneous inhibition of the PIK3CA and Yap cascades is extremely harmful for the *in vitro* growth of HCC and CCA cells.

## DISCUSSION

Mounting evidence indicates that activation of PI3K/AKT/mTOR and Yap signaling pathways is a driver oncogenic event in liver carcinogenesis [8, 9, 12, 13, 29]. In addition, a recent study showed that HCC samples with high AKT activation/phosphorylation also exhibit high levels of nuclear/activated Yap, implying a coordinated induction of the PI3K/AKT/mTOR and Yap cascades in HCC [21]. However, the functional crosstalk between the two signaling pathways in liver cancer has not been investigated to date. To the best of our knowledge, this is the first report showing that the cooperation of the PI3K and Yap cascades is sufficient to drive tumor development in the mouse liver.

In the current study, we found that overexpression of an activated form of PIK3CA led to the development of lipid-rich hepatocellular lesions that, however, were unable to undergo malignant transformation. Overexpression of Yap alone did not lead to any liver abnormality. In striking contrast, concomitant expression of PIK3CA and Yap resulted in rapid formation of liver tumors. This *in vivo* result strongly demonstrates the synergistic role of PIK3CA and Yap in the molecular pathogenesis of liver tumors. Clearly, the precise molecular mechanisms underlying liver tumor development induced by PIK3CA and Yap require further investigation. It is important to note that PIK3CA/Yap mice developed malignant lesions resembling histological features of HCC, CCA, and mixed HCC/CCA. Since we have previously proven that hydrodynamic gene delivery specifically targets mature hepatocytes [22, 30], the present results suggest that PIK3CA/Yap overexpression is sufficient not only to drive tumor development in the liver but also to promote dedifferentiation of hepatocytes into premalignant and malignant cells with hepatocellular and cholangiocellular features. Emerging data suggest that the process of dedifferentiation and reprogramming into different cell lineage(s), known as cellular plasticity, can contribute to the development of various tumor types [31, 32]. The cellular plasticity of hepatocytes with regard to carcinogenesis has been recently described in multiple investigations. For instance, it has been shown that mutant isocitrate dehydrogenase 1 (IDH1) promotes CCA development by stimulating hepatocyte dedifferentiation via silencing of HNF4A, the master regulator of hepatocyte identity [33]. In another study, it has been found that loss of the p53 tumor suppressor gene leads to the dedifferentiation of mouse hepatocytes into Nestin-positive progenitor-like cells, and these progenitor-like cells could be differentiated into HCC by activated Wnt/β-catenin or CCA by activated Notch signaling, respectively [34]. In addition, it has been shown that hepatocyte growth factor (HGF)/MET and epidermal growth factor (EGF)/EGF receptor (EGFR), two pivotal players in the normal and diseased liver, strongly affect the fate of hepatic progenitor cells [35]. In particular, MET activation induces hepatocyte differentiation, whereas EGFR promotes cholangiocyte specification and suppresses hepatocyte commitment via activation of the Notch cascade [35]. Furthermore, a recent study demonstrates that acute activation of Yap is sufficient to dedifferentiate mature hepatocytes into progenitor-like cells [36]. Based on the latter evidence, it is tempting to hypothesize that Yap promotes the dedifferentiation of hepatocytes in mouse livers co-injected with PIK3CA/Yap. These dedifferentiated hepatocytes might be subsequently transformed by the activated PI3K/AKT/mTOR cascade, as the PI3K/AKT/mTOR pathway might provide the necessary cell proliferative and metabolic requirement for tumor formation. Nevertheless, we cannot exclude that activation of the PI3K/AKT/mTOR cascade influences liver cell fate as well, since we have previously shown that overexpression of myristylated/activated AKT1 in the mouse liver leads to the development of both HCC and CCA [23]. In this regard, it is interesting to mention that some of the PIK3CA/AKT/mTOR targets, such as p-RPS6, FASN, and ACAC were almost exclusively upregulated in the lesions with hepatocellular features, whereas p-NDRG1 was mainly induced in cholangiocellular lesions. Although these findings require further investigation, the present data suggest that the PI3K/AKT/mTOR pathway might also promote the lineage commitment of liver cells by modulating the expression of selected target genes.

The molecular mechanisms responsible for the functional crosstalk between the PI3K and Yap cascades leading to liver tumor development remain unknown. Nonetheless, it is worthwhile mentioning that the *in vitro* studies conducted in the present investigation suggest a mutual regulation between the PI3K and Yap pathways in liver cancer. On the one hand, indeed, inactivation of PI3K by PIK75 resulted in the suppression of Yap and its target, CTGF, in HLF and EGI1 cells. On the other hand, suppression of Yap transcriptional activity triggered the downregulation of PIK3CA targets, NDRG1 and 4-EBP1, in the HLF cell line. Further investigations in multiple liver cancer cell lines are necessary to better understand the functional crosstalk between the PI3K and Yap cascades as well as to identify the crucial mediators involved in the interplay between these two oncogenic pathways.

Targeted therapy has opened up new prospects for biological therapy in human cancers, and it underscores the considerable significance of understanding genetic alterations and related molecular mechanisms underlying cancer initiation and development. Here, we described a novel mouse liver model induced by activated PIK3CA and Yap protooncogenes, which are frequently and concomitantly deregulated in HCC, CCA, and mixed HCC/CCA. This model could represent an ideal tool for mechanistic investigation of liver carcinogenesis and the preclinical studies evaluating therapies targeting the signaling pathways frequent activated in human liver cancers. Importantly, we found that concomitant inhibition of PIK3CA and Yap is highly detrimental for the growth of both HCC and CCA cell lines *in vitro*. Together with the *in vivo* findings, the latter data suggest that combined suppression of the PI3K/AKT/mTOR and Yap pathways might be highly beneficial for the treatment of liver cancer. Of note, recent studies have demonstrated the importance of Notch signaling in HCC and CCA molecular pathogenesis [30, 37, 38] as well as a major downstream effector of the Yap stimuli in the liver [24, 36]. As effective Yap inhibitors are missing, targeting Notch might be a plausible strategy to treat Yap-positive liver tumors. Several γ-secretase inhibitors (GSIs), the most commonly used small molecules against Notch, and anti-Notch specific antibodies have been developed and are currently tested in clinical trials for the treatment of solid tumors [39, 40]. Thus, the use of Notch inhibitors either alone or in association with PI3K/AKT/mTOR inhibitors might be an innovative and effective therapeutic approach for the treatment of human liver tumors characterized by the aberrant activation of the Yap and PI3K/AKT/mTOR pathways.

## MATERIALS AND METHODS

### Constructs and reagents

The constructs used for mouse injection, including pT3-EF1α-YapS127A, pT3-EF1α-YapS127AS94A, and pCMV/sleeping beauty transposase (SB), were described previously [22]. Human PIK3CA clone with H1047R mutation (PIK3CAH1047R) was obtained from Addgene (Plasmid 12524), and cloned into pT3-EF1α plasmid via the Gateway PCR cloning strategy (Invitrogen, Carlsbad, CA). Plasmids were purified using the Endotoxin free Maxi prep kit (Sigma-Aldrich, St. Louis, MO) before being injected into the mice.

### Hydrodynamic injection and mouse monitoring

Wild-type FVB/N mice were obtained from Charles River (Wilmington, MA). Hydrodynamic injection was performed as described [41]. In brief, 10μg pT3-EF5α-PIK3CAH1047R (with a HA tag) and pT3-EF5α-YapS127A (with a Flag tag) along with sleeping beauty transposase (SB) in a ratio of 25:1 were diluted in 2 ml saline (0.9% NaCl), filtered through 0.22 μm filter, and injected into the lateral tail vein of 6 to 8-week-old FVB/N mice in 5 to 7 seconds. Mice were housed, fed, and monitored in accordance with protocols approved by the Committee for Animal Research at the University of California, San Francisco.

### Immunohistochemical staining

Liver specimens were fixed in 4% paraformaldehyde and embedded in paraffin. Preneoplastic and neoplastic liver lesions were assessed by two board-certified pathologists (M.E. and F.D.) in accordance with the criteria by Frith et al. [42], as previously described in detail [43]. For immunohistochemistry, deparaffinized sections were incubated in 3% H_2_O_2_ dissolved in 1X phosphate-buffered saline (PBS) for 30 minutes to quench the endogenous peroxidase. For antigen retrieval, slides were microwaved in 10 mM citrate buffer (pH 6.0) for 12 minutes. Subsequently, slides were incubated with primary antibodies (Supplementary Table 1) overnight at 4°C. All the primary antibodies used in the present investigation were selected among those that were previously validated by the manufacturers for immunohistochemistry. The immunoreactivity was visualized with the Vectastain Elite ABC kit (Vector Laboratories, Burlingame, CA), using Vector NovaRED™ (Vector Laboratories) as the chromogen. Slides were counterstained with Mayer's hematoxylin. The specificity of primary antibody reactivity was confirmed by either omitting the primary antibody in the immunohistochemical procedure or, when available, by incubating for 2 hours at room temperature the primary antibody with its specific blocking peptide in a 1:2 dilution before adding the primary antibody to the slides. For mouse samples, levels of the investigated proteins were quantified semi-quantitatively. The intensity of immunostaining was defined with a scale from “–“ to “+++” (−, negative; +, weak; ++ moderate; +++, strong). At least 20 lesions per each mouse were evaluated. A summary of the immunohistochemical results in mouse livers is shown in Supplementary Table 2. In human specimens, immunoreactivity for PIK3CA was estimated semi-quantitatively: upregulation of PIK3CA was defined when immunolabeling for the latter protein was stronger in tumors when compared to corresponding surrounding non-neoplastic livers. Nuclear accumulation of the Yap protein was instead used to establish Yap activation. Double Hep Par1/CK7 immunolabeling in mixed HCC/CCA specimens was performed with the automated Leica Bond^tm^ staining system (Leica Biosystems, Wetzlar, Germany). Hep Par1 immunolabeling was revealed in red color using the Bond Polymer Refine Red Detection Kit (Leica Biosystems), whereas CK7 immunostaining was revealed in brown color by the Bond Polymer Refine Detection kit (Leica Biosystems).

### Western blot analysis

Liver cancer cell lines were lysed in lysis buffer [30 mM Tris (pH 7.5), 150 mM NaCl, 1% NP-40,0.5% Na deoxycholate, 0.1% SDS, 10% glycerol, and 2mM EDTA] containing the Complete Protease Inhibitor Cocktail (Roche Molecular Biochemicals, Indianapolis, IN). Protein concentrations were determined with the Bio-Rad Protein Assay Kit (Bio-Rad, Hercules, CA) using bovine serum albumin as standard. For Western blotting, aliquots of 40 μg were denatured by boiling in Tris-Glycine SDS Sample Buffer (Invitrogen, Carlsbad, CA), separated by SDS-PAGE, and transferred onto nitrocellulose membranes (Invitrogen) by electroblotting. Membranes were blocked in Pierce Protein-free Tween 20 Blocking Buffer (ThermoFisher Scientific, Waltham, MA) for 1 h and probed with the following primary antibodies: mouse monoclonal anti-Yap (Sigma-Aldrich, St. Louis, MO; cat. N. WH0010412M1-100UG; dilution 1:300), mouse monoclonal anti-CTGF (Santa Cruz Biotechnology, Santa Cruz, CA; cat. N. Sc-101586; dilution 1:300), mouse monoclonal anti-β-Actin (Sigma-Aldrich; cat. N. A1978-200UL; dilution 1:5000), rabbit monoclonal anti-phospho-NDRG1 (Cell Signaling Technology Inc., Danvers, MA; cat. N. 5482; dilution 1:300), and rabbit monoclonal anti-phospho-4EBP1 (Cell Signaling Technology; cat. N. 2855; dilution 1:300). Each primary antibody was followed by incubation with horseradish peroxidase-secondary antibody diluted 1:5000 for 1 h and then revealed with the Super Signal West Pico (Pierce Chemical Co., New York, NY). Equal loading was assessed by reversible Ponceau Red Staining (Sigma-Aldrich, St. Louis, MO) and β-Actin immunoblotting.

### Human liver tissue specimens

A collection of formalin-fixed, paraffin-embedded HCC (n=54), CCA (n=42), and mixed HCC/CCA (n=16) samples was used in the present study. The clinicopathological features of liver cancer patients are summarized in Supplementary Table 3-5. HCC specimens were kindly provided by Dr Snorri S. Thorgeirsson (National Cancer Institute, Bethesda, MD, USA) and collected at the Institute of Pathology of the University of Greifswald (Greifswald, Germany), whereas CCA specimens were collected in the University of Greifswald, and the mixed HCC/CCA specimens in the Department of Digestive and Hepatobiliary Surgery, Henri Mondor Hospital (Créteil, France). The mixed HCC/CCA consisted of the three entities previously described by Allen and Lisa [44]: (a) separate masses composed of either hepatocellular or cholangiocellular components; (b) contiguous but independent masses of hepatocellular and cholangiocellular components; and (c) an intimate intermingling of hepatocellular and glandular elements. Institutional Review Board approval was obtained at the participating hospitals and the National Institutes of Health.

### Cell lines and treatments

The human HCC cell lines HLF and SK/Hep1 as well as human CCA cell line EGI1 cell line were grown in Dulbecco's Modified Eagle's Medium supplemented with 10% fetal bovine serum and used for the experiments. For the treatment with chemical inhibitors, the three cell lines were plated at 2.0 × 10^3^/well in 96-well plate and grown for 12 hours. After 24-hour serum deprivation, PIK75 (Selleck Chemicals, Houston, TX) and/or Verteporfin (Yap-TEAD disruptor; VWR International GmbH, Darmstadt, Germany) were added to the medium at 1 μM, and 2 μM final concentration, respectively, and cells incubated for 48 hours. To assess cell proliferation, the three cell lines were plated at the concentration of 2.0 × 10^3^/well in 96-well plates, allowed to attach and adjust for the next 12 and grown for additional 48 hours. The proliferation was assessed at 48 hours with the BrdU Cell Proliferation Assay Kit (Cell Signaling Technology, Danvers, MA) by measuring the absorbance at 450 nm following the manufacturer's protocol. To measure apoptosis, cell lines were plated at the concentration of 2.0 × 10^3^/well in 96-well plates, incubated for 12 hours, and then subjected to 24-hour serum deprivation. Cell lines continued to grow in serum-free medium for additional 48 hours. Apoptosis was assessed at the latter time point with the Cell Death Detection Elisa Plus Kit (Roche Molecular Biochemicals, Indianapolis, IN) by measuring the absorbance at 405 nm, following the manufacturer's instructions.

### Statistical analysis

Data analysis was performed with SPSS 19 (IBM SPSS, Armonk, NY, USA). All data are presented as Means± SE. Statistical differences among the various groups were assessed with the Tukey-Kramer's test. P values < 0.05 were considered statistically significant.

## SUPPLEMENTARY MATERIALS, FIGURES AND TABLES


