# Supplementary material for: Co-activation of PIK3CA and Yap promotes development of hepatocellular and cholangiocellular tumors in mouse and human liver
Source: Oncotarget. 2015 Mar 12;6(12):10102–15. doi: 10.18632/oncotarget.3546 (PMC4496343; doi:10.18632/oncotarget.3546)
Supplement: Supplementary file 1 [file oncotarget-06-10102-s001.pdf]

Co-activation of PIK3CA and Yap promotes development of hepatocellular and cholangiocellular tumors in mouse and human liver

Supplementary Material

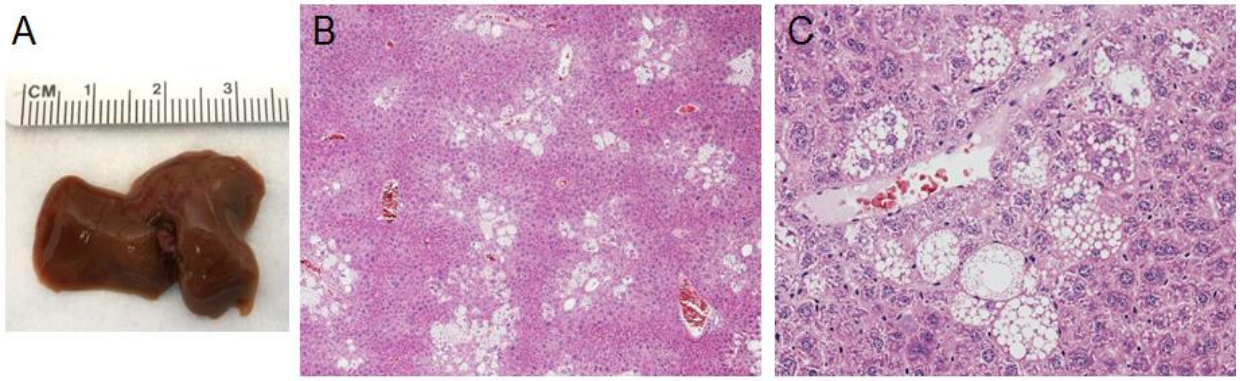

Supplementary Figure 1: Lipid accumulation in hepatocytes but absence of liver tumor development in mice injected with the PIK3CAH1047R plasmid. (A) Macroscopic appearance of a PIK3CAH1047-injected liver showing the absence of gross alterations. (B) Microscopically, at lower magnification, numerous clear-cell hepatocytes around the hepatic veins characterize the liver parenchyma of PIK3CAH1047R mice 40 weeks post hydrodynamic injection, as assessed by hematoxylin and eosin staining. (C) At higher magnification, these hepatocytes show an enlarged cytoplasm due to increased lipid storage. Original magnification: 40X in B; 200X in C.

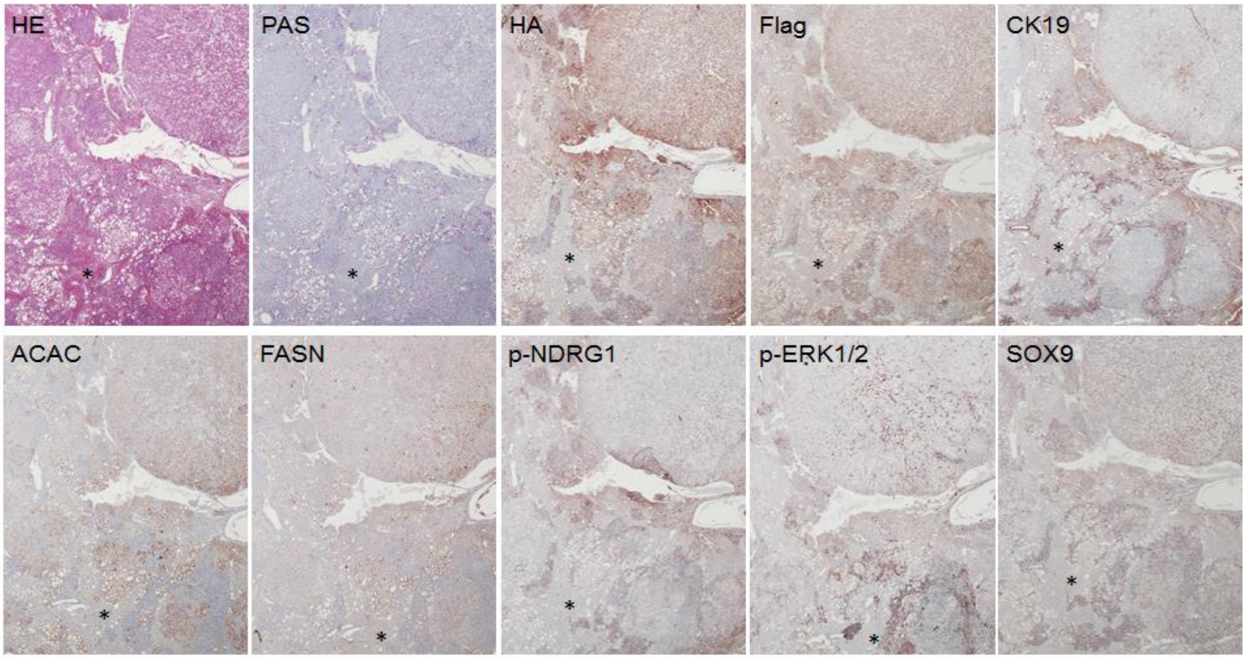

Supplementary Figure 2: Summary of the immunohistochemical patterns detected in the lesions of PIK3CA/Yap mice 12 weeks post hydrodynamic injection. HE staining shows the presence of numerous neoplastic lesions with hepatocellular and/or cholangiocellular features occupying the vast majority of the liver parenchyma at this time point. Limited, residual areas of normal liver are present in PIK3CA/Yap livers (one of these areas is indicated by the asterisk). Of note, hepatocellular lesions display glycogen accumulation (as indicated by the positiveness to PAS staining), whereas cholangiocellular lesions are PAS negative. At the immunohistochemical level, these tumors are homogeneously immunoreactive for HA-tagged PIK3CAH1047R (HA) and Flag-tagged Yap (Flag), underlying their origin from doubly-transfected cells. As expected, CK19 immunolabeling was detected only in the cholangiocellular fraction of the lesions. Importantly, levels of the lipogenic proteins acetyl-CoA carboxylase (ACAC) and fatty acid synthase (FASN) were elevated only in hepatocellular lesions, whereas the mTORC2 surrogate marker, phosphorylated N-Myc downregulated gene 1 (p-NDRG1), and phosphorylated/activated extracellular activated kinase 1/2 (p-ERK1/2) proteins were strongly induced only in cholangiocellular lesions. In addition, immunoreactivity for SOX9 (a marker of Notch cascade activation) was evident both in the hepatocellular and cholangiocellular component. Of note, the area of normal liver does not show immunoreactivity for the investigated proteins, besides a very weak staining for ACAC. In the same area, positive immunolabeling for CK19 is limited to normal biliary epithelial cells. Serial sections are shown as an example in the present figure. Original magnification: 40X. Abbreviation: HE, hematoxylin and eosin staining. A more detailed description of the lesions developed in PIK3CA/Yap mice and their immunohistochemical pattern is reported in Figure 1-4 and Supplementary Table 2.

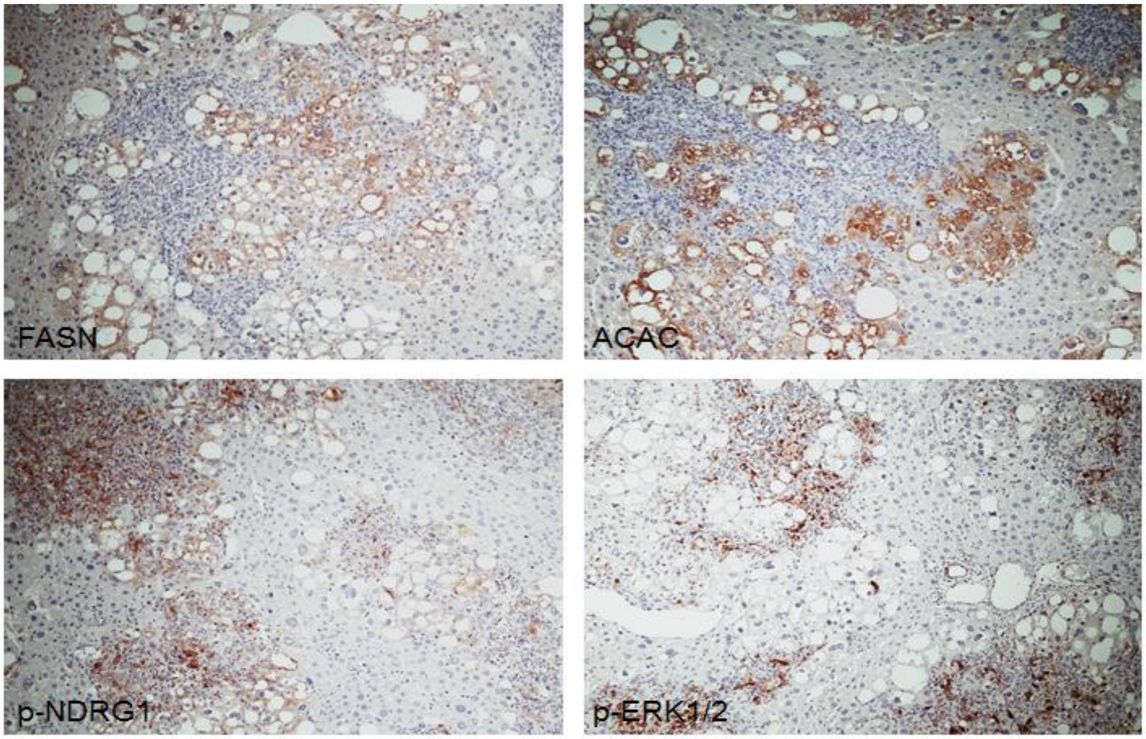

Supplementary Figure 3: Different staining patterns for mTOR targets and ERK proteins in hepatocellular and cholangiocellular components of mixed tumors developed in PIK3CA/Yap mice. While lipogenic proteins (FASN and ACAC) showed immunolabeling almost exclusively in tumor cells with hepatocellular differentiation, immunoreactivity for phosphorylated NDRG1 (p-NDRG1; a surrogate marker of mTORC2 activation) and phosphorylated/activated ERK1/2 (p-ERK1/2) proteins characterize the cholangiocellular part of the tumor. Magnification: 100X.

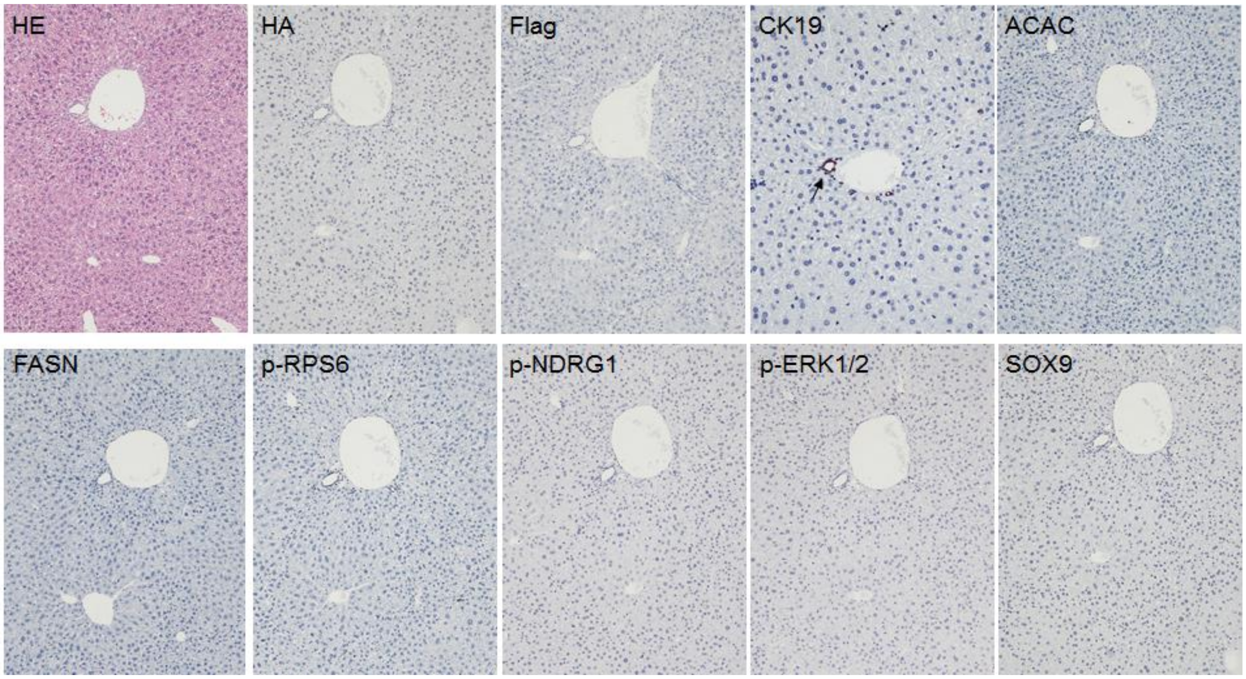

Supplementary Figure 4: Summary of the immunohistochemical patterns detected in the livers of mice injected with the empty vector 12 weeks post hydrodynamic injection. HE staining shows the absence of morphological alterations in the livers of these mice. At the immunohistochemical level, immunolabeling for HA-tag, Flag-tag, acetyl-coA carboxylase (ACAC), fatty acid synthase (FASN), phosphorylated/activated ribosomal protein S6 (p-RPS6), phosphorylated N-Myc downregulated gene 1 (p-NDRG1), phosphorylated/activated extracellular activated kinase 1/2 (p-ERK1/2), and SOX9 proteins is very weak or absent. As expected, positive immunoreactivity for CK19 is limited to normal biliary epithelial cells (indicated by an arrow). Serial sections are shown as an example in the present figure. Equivalent results were obtained in wild-type, not injected livers (not shown). Original magnification: 200X for CK19; 100X for all the other proteins tested. Abbreviation: HE, hematoxylin and eosin staining.

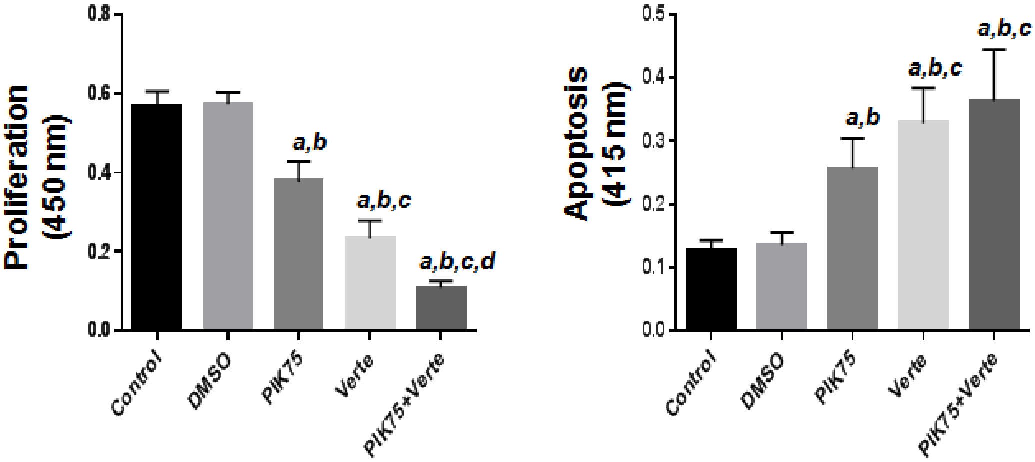

Supplementary Figure 5: Suppression of PIK3CA and Yap activity via specific inhibitors is highly detrimental for the growth of the human SK/Hep1 hepatocellular carcinoma (HCC) cell line. Treatment with the PIK3CA inhibitor, PIK75 (1 μM), or the Yap/TEAD disruptor, Verteporfin (Verte; 2 μM) decreased proliferation (left panel) and induced apoptosis (right panel) in the SK/Hep1 HCC cell line when compared with control (untreated) and DMSO (solvent) treated cells. Importantly, combined administration of PIK75 and Verteporfin further decreased the proliferation rate of SK/Hep1 cells without further augmenting apoptosis. Each bar represent mean ± SD of three independent experiments conducted in triplicate. Tukey-Kramer's test: P at least < 0.05 *a*, versus control (untreated cells); *b*, versus DMSO (solvent); *c,* versus PIK75; *d*, versus Verteporfin. Abbreviation: Verte, Verteporfin.

Supplementary Table 1: List of the primary antibodies used for immunohistochemistry

Protein	Antibody (and catalog number)	Epitope mapping
Phospho-AKT	Rabbit monoclonal (13038)	Serine 473†
Flag-Tag	Rabbit polyclonal (2368)	Flag-Tag†
HA-Tag	Mouse monoclonal (2367)	HA-Tag†
CK19	Rat monoclonal (TROMAIII)	Not available††
Phospho-ERK1/2	Rabbit monoclonal (4370)	pThr202/Tyr204†
ACAC	Rabbit polyclonal (3662)	Residues around Lys557†
Phospho-RPS6	Rabbit monoclonal (2855)	Ser240/244†
FASN	Mouse monoclonal (610962)	Amino acids 9-202*
Phospho-mTOR	Rabbit monoclonal (2976)	Ser2448†
Phospho-NDRG1	Rabbit monoclonal (5842)	Thr346†
SOX9	Rabbit polyclonal (sc-20095)	Amino acids 407-496**
Notch2	Rabbit monoclonal (5732)	Residues around Ala2378†
CK7	Mouse monoclonal (M7018)	Not available†††
Hep Par1	Mouse monoclonal (M7158)	Not available†††
Ki67	Rabbit polyclonal (IHC-00375)	Residues 1650-1700††††

† Provided by Cell Signaling Technology Inc. (Danvers, MA).

†† Provided by Developmental Studies Hybridoma Bank (Iowa City, IA).

††† Provided by Dako Deutschland (Hamburg, Germany).

†††† Provided by Bethyl Laboratories, Inc. (Montgomery, TX).

* Provided by BD Biosciences (Franklin Lakes, NJ).

** Provided by Santa Cruz Biotechnology (Santa Cruz, CA).

Supplementary Table 2: Summary of the immunohistochemical patterns detected in wild-type livers (Wild-type), livers injected with empty vector (Empty vector) as well as in hepatocellular and cholangiocellular lesions (both preneoplastic and neoplastic) developed in PIK3CA/Yap mice

Protein name	Wild-type	Empty vector	PIK3CA/YAP (hepatocellular lesions)	PIK3CA/YAP (cholangiocellular lesions)
Flag-tag (Yap)	-	-	++	+++
HA-tag (PIK3CA)	-	-	+++	+++
p-AKT	-	-	+++	+++
p-mTOR	-	-	++	++
FASN	+/-	+/-	+++	+/-
ACAC	+/-	+/-	+++	+/-
p-RPS6	+/-	+/-	+++	+
p-NDRG1	-	-	+	+++
p-ERK1/2	-	-	+/-	+++
Notch2	-	-	++	++
SOX9	-	-	+++	+++
CK19	-*	-*	-*	+++

The intensity of immunostaining was defined semi-quantitatively with a scale from "–" to "+++" (-, negative; +, weak; ++, moderate; +++, strong). The asterisks (*) indicates that CK19 immunoreactivity is limited to normal biliary epithelial cells in wild-type livers, livers injected with empty vector, and hepatocellular lesions from PIK3CA/Yap mice.

Supplementary Table 3: Clinicopathological features of hepatocellular carcinoma (HCC) patients

Variables	
No. of patients	54
Male	44
Female	10
Etiology	
HBV	22
HCV	15
Ethanol	12
NA	5
Cirrhosis	
+	42
-	12
Tumor size	
> 3 cm	39
< 3 cm	15
Edmondson and Steiner grade	
I	5
II	15
III	22
IV	12
Alpha-fetoprotein secretion	
> 300 ng/ml of serum	30
< 300 ng/ml of serum	24

Abbreviation: NA, not available

Supplementary Table 4: Clinicopathological features of cholangiocarcinoma (CCA) patients

Variables	
No. of patients	42
Male	34
Female	8
Etiology	
HBV	4
HCV	10
Hepatolithiasis	6
PSC	4
NA	18
Tumor differentiation	
Well	16
Moderately	14
Poorly	12

Abbreviations: NA, not available; PSC, primary sclerosing cholangitis.

Supplementary Table 5: Clinicopathological features of mixed hepatocellular/cholangiocarcinoma (HCC/CCA) patients

Variables	
No. of patients	16
Male	15
Female	1
Etiology	
HBV	2
HCV	5
HCV & Alcohool	1
Alcohol	1
Alcohol and metabolic syndrome	1
NASH and metabolic syndrome	2
NA	4

Abbreviations: NA, not available; NASH, non-alcoholic steatohepatitis.
